# Whole-Exome sequencing analysis identified TMSB10/TRABD2A locus to be associated with carfilzomib-related cardiotoxicity among patients with multiple myeloma

**DOI:** 10.3389/fcvm.2023.1181806

**Published:** 2023-06-20

**Authors:** Marwa Tantawy, Guang Yang, Raghunandan Reddy Algubelli, Gabriel DeAvila, Samuel M. Rubinstein, Robert F. Cornell, Michael G. Fradley, Erin M. Siegel, Oliver A. Hampton, Ariosto S. Silva, Daniel Lenihan, Kenneth H. Shain, Rachid C. Baz, Yan Gong

**Affiliations:** ^1^ Department of Pharmacotherapy and Translational Research and Center for Pharmacogenomics and Precision Medicine, College of Pharmacy, University of Florida, Gainesville, FL, United States; ^2^ Department of Pharmacology, Feinberg School of Medicine, Northwestern University, Chicago, IL, United States; ^3^ Department of Malignant Hematology, H. Lee Moffitt Cancer Center & Research Institute, Tampa, FL, United States; ^4^ Department of Medicine, Division of Hematology, University of North Carolina, Chapel Hill, NC, United States; ^5^ Department of Medicine, Division of Hematology and Oncology, Vanderbilt University Medical Center, Nashville, TN, United States; ^6^ Cardio-Oncology Center of Excellence, Division of Cardiology, Department of Medicine, Perelman School of Medicine at the University of Pennsylvania, Philadelphia, PA, United States; ^7^ Department of Cancer Epidemiology, H. Lee Moffitt Cancer Center & Research Institute, Tampa, FL, United States; ^8^ Department of Biostatistics and Bioinformatics, H. Lee Moffitt Cancer Center & Research Institute. Tampa, FL, United States; ^9^ Department of Cancer Physiology, H. Lee Moffitt Cancer Center & Research Institute, Tampa, FL, United States; ^10^ Cape Cardiology Group, Saint Francis Medical Center, Cape Girardeau, MO, United States; ^11^ Cancer Control and Population Sciences, UF Health Cancer Center, University of Florida, Gainesville, FL, United States

**Keywords:** cardio-oncology, proteosome inhibitors, Multiple Myeloma, carfilzomib, cardiotoxcity, whole exome sequencing

## Abstract

**Background:**

Proteasome inhibitor Carfilzomib (CFZ) is effective in treating patients with refractory or relapsed multiple myeloma (MM) but has been associated with cardiovascular adverse events (CVAE) such as hypertension, cardiomyopathy, and heart failure. This study aimed to investigate the contribution of germline genetic variants in protein-coding genes in CFZ-CVAE among MM patients using whole-exome sequencing (WES) analysis.

**Methods:**

Exome-wide single-variant association analysis, gene-based analysis, and rare variant analyses were performed on 603,920 variants in 247 patients with MM who have been treated with CFZ and enrolled in the Oncology Research Information Exchange Network (ORIEN) at the Moffitt Cancer Center. Separate analyses were performed in European Americans and African Americans followed by a trans-ethnic meta-analysis.

**Results:**

The most significant variant in the exome-wide single variant analysis was a missense variant rs7148 in the thymosin beta-10/TraB Domain Containing 2A (*TMSB10/TRABD2A*) locus. The effect allele of rs7148 was associated with a higher risk of CVAE [odds ratio (OR) = 9.3 with a 95% confidence interval of 3.9—22.3, *p* = 5.42*10^−7^]. MM patients with rs7148 AG or AA genotype had a higher risk of CVAE (50%) than those with GG genotype (10%). rs7148 is an expression quantitative trait locus (eQTL) for *TRABD2A* and *TMSB10*. The gene-based analysis also showed *TRABD2A* as the most significant gene associated with CFZ-CVAE (*p* = 1.06*10^−6^).

**Conclusions:**

We identified a missense SNP rs7148 in the *TMSB10/TRABD2A* as associated with CFZ-CVAE in MM patients. More investigation is needed to understand the underlying mechanisms of these associations.

## 1. Introduction

Multiple Myeloma (MM) is a malignancy of the plasma cells. According to yearly incidence rates and prevalence figures, it ranks third among hematologic cancers in the United States (1). Proteasome inhibitors (PIs) are one of the most effective drugs for the treatment of MM and are the backbone therapies for MM treatment (2). Three PIs have been approved by the United States Food and Drug Administration: bortezomib, carfilzomib, and ixazomib (3). Carfilzomib (Kyprolis®) is a second-generation, irreversible PI approved for treating relapsed and refractory MM due to its survival benefit and overall response rate in refractory MM patients (4, 5). The National Comprehensive Cancer Network (NCCN) Guideline recommends carfilzomib-based therapy for newly diagnosed MM patients with pre-existing neuropathy or high-risk patients and patients with relapsed and refractory disease (6). Despite its effectiveness, carfilzomib (CFZ) has been shown to have significant cardiovascular adverse events (CVAE) (20%–25%), including 7.2% incident HF, in the clinical trials that excluded patients with pre-existing cardiovascular disorders (7). In the previous two meta-analyses studies, CFZ was associated with a high incidence of CVAE (8%–18%) (2, 3) including hypertension, heart failure, cardiomyopathy, and arrhythmia (8, 9). The rate of CVAE was even higher (∼50%) in an observational study when MM patients with pre-existing cardiovascular conditions were not excluded (10). A severe clinical implication developed from this cardiotoxicity is treatment interruption, which could lead to disease progression (11).

Studies have shown that early detection of and early intervention for cardiotoxicity induced by other therapies (i.e., anthracyclines, Trastuzumab) can improve long-term outcomes (12, 13). Therefore, stratifying patients before the carfilzomib treatment might provide opportunities for early intervention to optimize patient outcomes. Pharmacogenomics, or the identification of genetic determinants of drug response and adverse effects, is a tool that has been useful in individualizing medication therapy (14, 15). This study aims to identify germline genetic variants in protein-coding genes associated with CFZ-CVAE in MM patients using whole-exome sequencing (WES) analysis.

## 2. Material and methods

### 2.1. Patients

Patients included in this study were admitted to the Moffitt Cancer Center's Total Cancer Care (TCC) Protocol with IRB approval (MCC#14690; Advarra IRB Pro00014441) (16). Patients consented to contribute blood specimens and medical information for research purposes in collaboration with the Oncology Research Information Exchange Network (ORIEN), a network of seventeen cancer centers that have agreed to implement a common TCC biospecimen collection protocol to follow patients throughout their lifetime (17). Clinical and epidemiological data were collected for select TCC-consented patients, and the molecular data were produced as described below. This study included a total of 247 patients who have been diagnosed with MM and treated with carfilzomib at the H. Lee Moffitt Cancer Center and have germline DNA WES data available through ORIEN. This study was also approved by the University of Florida Institutional Review Board (IRB202003031).

### 2.2. Cardiovascular adverse events

We queried the electronic health records data of Moffitt's health research informatics platform to determine if a CVAE had occurred after initiation of carfilzomib treatment. We used the International Classification of Disease (ICD) revisions 9 and 10 to define cardiovascular events. A complete list of the ICD-9 and -10 codes used is listed in the supplementary materials Supplementary Table S1. Moffitt's Pentecost Myeloma Research Center clinical database was utilized to identify if the eligible TCC patients receiving carfilzomib as part of their treatment had one of these cardiac events between the start and end date. We performed a manual chart review on 10% of patients to verify the accuracy of the billing records in terms of the definition of the CVAE. All records reviewed for cardiovascular adverse events matched the determination by the billing codes.

### 2.3. Whole-Exome sequencing and quality control

Germline DNA was extracted from peripheral blood samples with buffy coat using the QIAsymphony SP instrument (QIAGEN, Hilden, Germany) following standard protocols implemented across the ORIEN. The WES of germline DNA was performed for each patient using SeqCap EZ Exome Enrichment Kit v3.0 (Roche NimbleGen, Pleasanton, CA) or xGen Exome Hybridization Panel with supplement probes (integrated Data Technologies, Inc., Coralville, IA), with 100 × coverage. Capture kits covered variants for limited regions; each captured library was loaded onto Illumina-HiSeq 4,000 (Illumina, San Diego, CA). Over 26,000 protein-coding genes were sequenced. The raw sequencing data underwent a rigorous analysis pipeline for alignment, variant calling, quality control steps, and annotation algorithms (18–20). Before the genetic association analysis, the WES data underwent additional quality control steps: variant call rate > 95%, sample call rate > 95%, sex check, and Hardy Weinberg Equilibrium analysis. Principle component analysis was performed on a subset of variants after more stringent quality control steps [variant rate and sample call rate > 99% and minor allele frequency (MAF) >10%] to evaluate the genetic ancestry of these patients.

Germline WES data was available on 605,446 variants in 247 patients. Of the 247 patients, 228 were genetically clustered with individuals of European ancestry, and 19 were clustered with African ancestry. A total of 603,920 variants passed the quality control steps and were included in the WES analysis.

### 2.4. Whole-Exome sequencing data analysis

All association analyses were performed in genetically clustered European Americans and African Americans separately, adjusting for age, gender, and principal components for ancestry. Trans-ethnic meta-analyses were then performed to combine the results from both groups.

Exome-wide association analysis of single variants with a MAF ≥ 1% was performed to estimate the odds ratio (OR) and 95% confidence interval (CI) for each variant on chromosomes 1–22 for the development of CFZ-CVAE using multivariable logistic regression assuming an additive model of inheritance using PLINK (21). All variants with *p* < 5*10^−8^ were considered statistical significance. Variants with *p* < 5*10^−4^ were considered suggestive (22).

Following the exome-wide association analysis, the summary statistics were functionally annotated using Functional Mapping and Annotation of Genome-Wide Association Studies (FUMA GWAS) for a gene-based and gene set analysis to recognize potential genes of interest (23). The Genotype-Tissue Expression (GTEx) database was queried to identify tissue-specific gene expression and regulation. ANNOVAR (24) was used to annotate the genetic variants appropriately.

Rare variants analysis was performed using the sequence kernel association test (SKAT) using the SKAT package (25, 26) to evaluate the association of the joint effect of multiple rare variants (MAF < 1%) with CFZ-CVAE.

### 2.5. Ingenuity pathway analysis (IPA)

Functional assignment and pathway analysis of the association results was performed on the top variants from the WES analyses (*p* < 0.001). IPA uses a network generation algorithm to create multiple networks and uses hypergeometric distribution to create scores for each network (27). The statistical significance level is generated using Fisher's Exact test. Any pathway enriched by genes more than by chance would be statistically significant.

## 3. Results

### 3.1. Patients characteristics

Overall, 247 MM patients were included in the analysis. The mean age was ∼59 years, and 57% were men. Thirty-eight (15.5%) developed CVAE after initiation and during carfilzomib-based therapy. Table 1 summarizes the characteristics of the 38 patients who developed CVAE and the 209 who did not. The baseline demographics and medical history were similar between the two groups of patients. A total of 228 (92.3%) patients were genetically clustered with individuals of European ancestry (EA), and 19 clustered with individuals of African ancestry (AA). The baseline characteristics and medical history of the 228 EA MM patients were summarized in Supplementary Table S2.

**Table 1:** Demographics and clinical characteristics of patients.

**Characteristics**	**Overall (*n* = 247)**	**CVAE (*n* = 38)**	**No CVAE (*n* = 209)**	***P***
**Age (years)**	58.9 ± 9.8	60.6 ± 9.4	58.6 ± 9.8	0.27
**Sex (male)**	141 (57.1%)	21 (55.3%)	120 (57.4%)	0.81
Race				
European American	228 (92.3%)	35 (92.1%)	193 (92.3%)	0.96
African American	19 (7.7%)	3 (7.9%)	16 (7.7%)	
Medical History				
Hyperlipidemia	78 (31.6%)	15 (39.4%)	63 (30.1%)	0.26
Hypertension	130 (52.6%)	25 (65.8%)	105 (50.2%)	0.077
Diabetes	22 (8.9%)	6 (15.8%)	16 (7.7%)	0.11
Ischemic Heart Disease	5 (2.0%)	1 (2.6%)	4 (1.9%)	0.77
Myocardial Infarction	12 (4.9%)	3 (7.9%)	9 (4.3%)	0.34

Continuous variables were summarized as mean ± standard deviation, and categorical variables were presented as numbers (%). *P* values shown were from a *t*-test for continuous variables and chi-squared test for categorical variables. CVAE: cardiovascular adverse events.

### 3.2. Exome-wide common variant analysis

**Figure 1:**
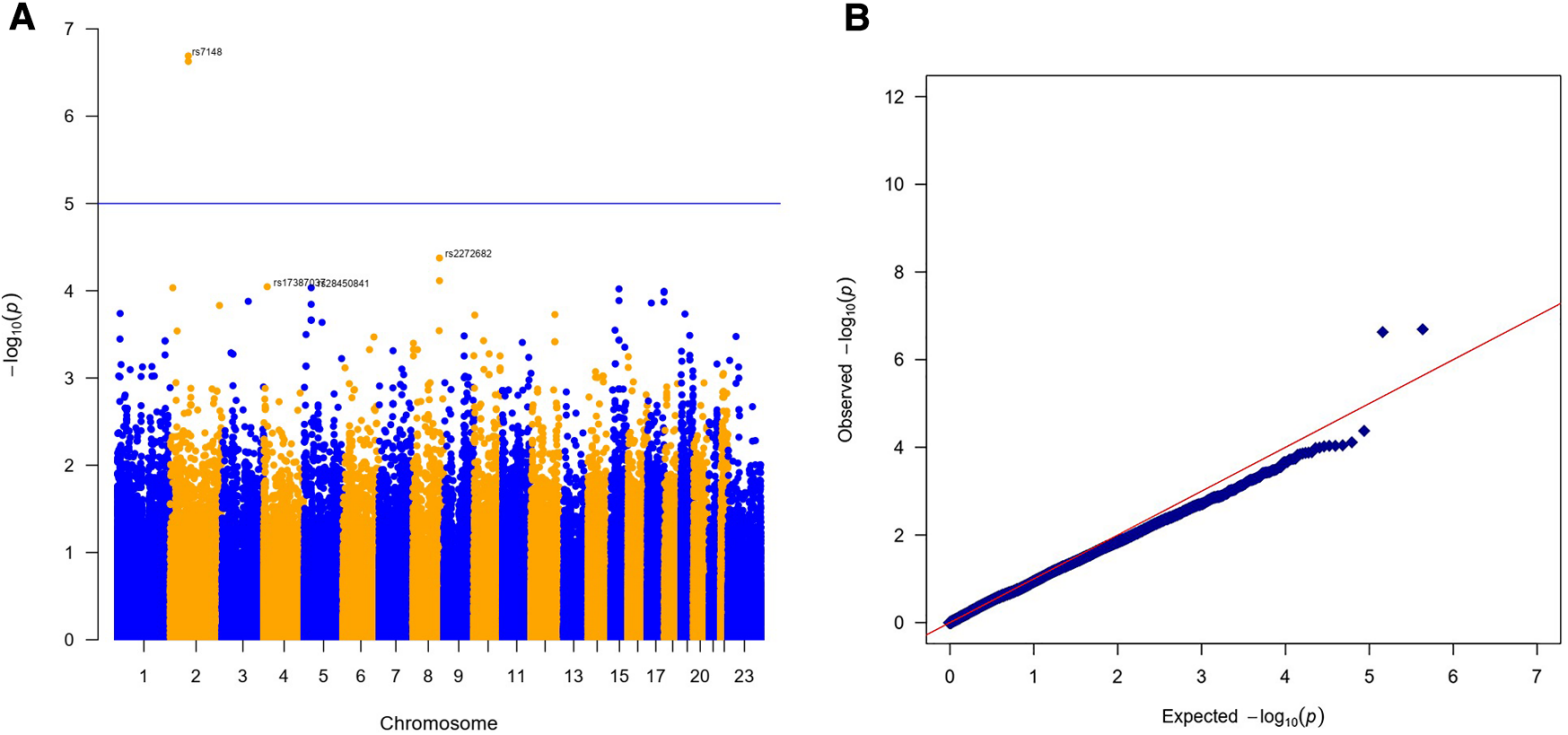
Results of exome-wide association analysis in European Americans. (**A**) Manhattan plot representing the association between single-nucleotide polymorphism (SNP) genotype and CVAE in MM patients. (**B**) The quantile-quantile (QQ) plot of association results.

**Figure 2:**
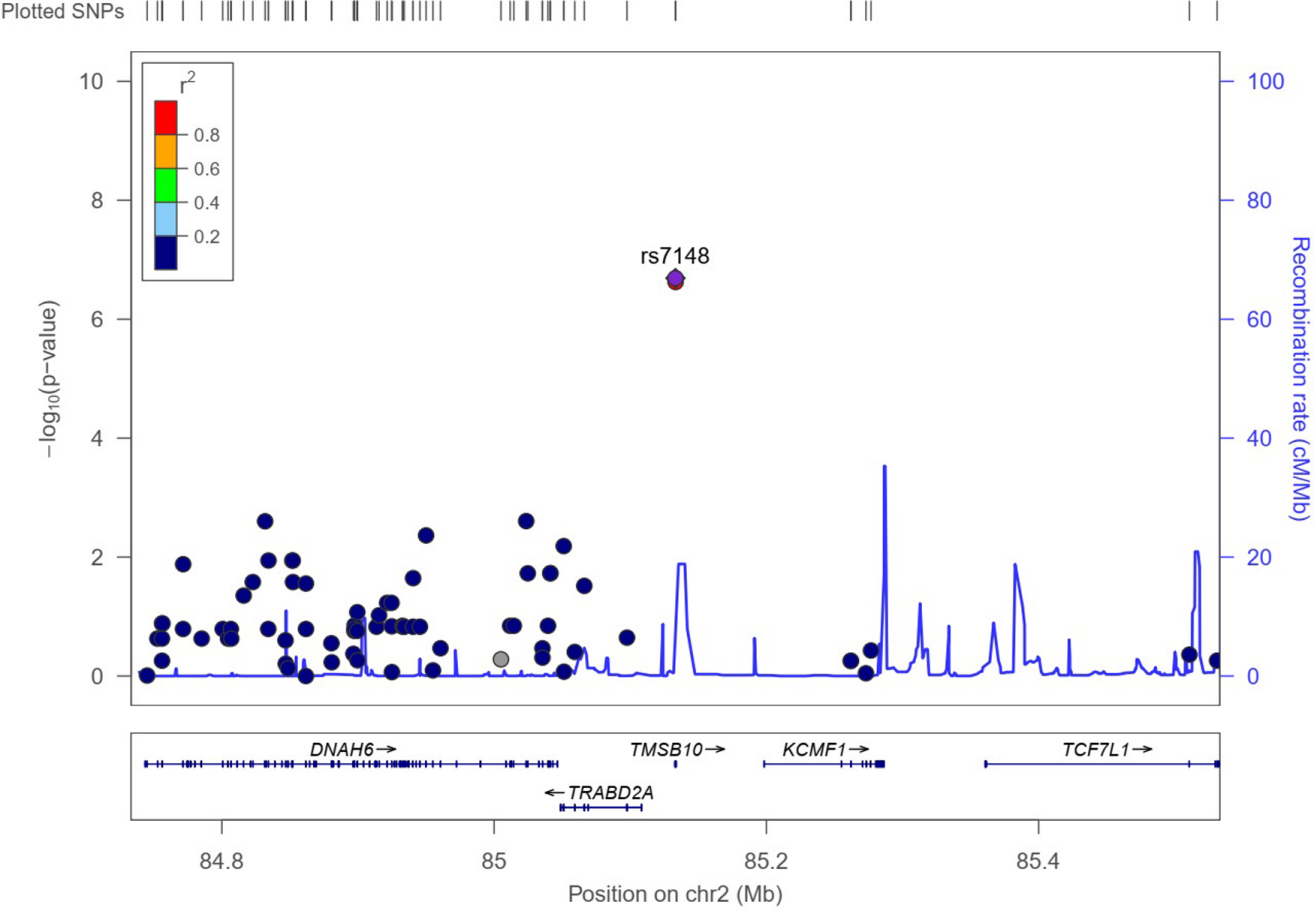
Regional association plot for variant rs7148. The X-axis represents a 1Mb region, 500 kb on either side of the variant, and the y-axis shows –the log_10_ *P*-value for individual SNPs. Pairwise LD (r^2^) with the variant is based on 1,000 Genome phase 3 v5 European reference samples and described using the color scale in the bar. The bottom panel shows the genes located within the region.

The results of the exome-wide association analysis of the common variants in the EA patients are summarized in the Manhattan plot (Figure 1A) and the QQ plot (Figure 1B). None of the variants were genome-wide significant. However, eleven SNPs from five loci reached the suggestive significance level with *p* < 5*10^−4^ (Table 2). The top SNP rs7148 is a missense variant in the thymosin beta-10 (*TMSB10)* gene, with OR of 9.33% and 95% CI of 3.90 – 22.35 (*p* = 5.42*10^−07^) (Table 2, Figure 2). The minor allele frequency rs7148 was ∼7% in EA patients. Amongst patients with the rs7148 variant, 50% (99/198) of patients with the AG or AA genotype developed a CVAE compared with 10.1% (20/198) with the GG genotype (p < 0.0001). GTEx analysis revealed that the rs7148 A allele was associated with higher TraB Domain Containing 2A (TRABD2A) gene expression in the left ventricle tissue with a *p*-value 1.9 × 10^−7^ (Supplementary Figure S1). The second most significant SNP, rs12471929, is an intronic variant in the *TMSB10* gene that is in perfect linkage disequilibrium (LD) (*r*^2^ = 1, D'=1) with rs7148, with OR of 8.95 (3.78–21.18), *p* = 6.17*10^−7^ (Table 2, Figure 2). A few other SNPs in LD with rs7148 in the 1,000 genomes database are shown in Supplementary Table S3.

**Table 2:** Top SNPs in the WES analysis of CFZ-CVAE in the European American patients.

**CHR**	**SNP**	**BP**	**Gene**	**A1**	**A2**	**MAF**	**OR**	**95% CI**	***P***	**dbSNP functional annotation**	**MAF CVAE**		**MAC CVAE**	
											**No**	**Yes**	**No**	**Yes**
2	rs7148	85,13,3216	TMSB10	A	G	0.068	9.33	3.90–22.35	5.42E-07	Missense	0.039	0.229	15	16
2	rs12,47,1929	85,13,3320	TMSB10	T	C	0.070	8.95	3.78–21.18	6.17E-07	Intronic	0.039	0.243	15	17
5	rs28,45,0841	32,09,3210	PDZD2	T	C	0.077	5.25	2.38–11.58	3.91E-05	Intronic	0.052	0.214	20	15
5	rs22,91,113	32,07,4509	PDZD2	A	G	0.057	6.17	2.52–15.15	7.05E-05	Synonymous	0.036	0.171	14	12
4	rs17,38,7037	15,99,2783	PROM1	C	A	0.057	5.84	2.44–139.5	7.32E-05	Intronic	0.036	0.171	14	12
2	rs75,34,8511	11,73,8951	GREB1	T	C	0.037	8.30	2.90–23.77	8.08E-05	Synonymous	0.021	0.129	8	9
8	rs22,72,682	12,60,49443	WASHC5	C	T	0.094	3.83	1.95–7.49	9.26E-05	Intronic	0.065	0.257	25	18
8	rs11,54,2889	12,60,44527	WASHC5/SQLE	T	C	0.059	5.01	2.23–11.24	9.50E-05	Synonymous	0.034	0.200	13	14
5	rs37,33,720	32,08,7808	PDZD2	C	G	0.072	4.90	2.20–10.87	9.55E-05	Synonymous	0.049	0.200	19	14
5	rs10,06,6063	32,09,0294	PDZD2	A	G	0.072	4.90	2.20–10.87	9.55E-05	Missense	0.049	0.200	19	14
5	rs16,88,9442	32,09,3070	PDZD2	A	G	0.072	4.90	2.20–10.87	9.55E-05	Intronic	0.049	0.200	19	14

WES, whole exome sequencing; CFZ-CVAE, carfilzomib-related cardiovascular adverse events; Chr., chromosome; SNP, single nucleotide polymorphism; BP: base pair. A1: minor allele; A2: major allele; MAF, minor allele frequency; OR: odds ratio; CI, confidence interval, MAC, minor allele counts.

Among the other SNPs with a suggestive level of significance were: five SNPs in the *PDZD2* gene, which encodes PDZ Domain Containing Protein 2; an intronic variant in the Prominin 1, CD133 (*PROM1*) gene; a synonymous variant on *GREB1* (Growth Regulating Estrogen Receptor Binding 1) gene on Chromosome 2, and two variants in *WASHC5/SQLE* gene on Chromosome 8 (Table 2). The minor allele frequencies, allele counts, and Hardy Weinberg Equilibrium test results by CVAE status are summarized in Supplementary Table S4.

We also performed an exploratory analysis on AA patients. No SNPs reached statistical significance as a result of the small sample size. The four SNPs with nominal significance (*p* < 0.05) are shown in Supplementary Table S5. The two most frequent SNPs in EA (rs7148, rs1247192) were not observed in AA patients (MAF = 0).

The top SNPs in the trans-ethnic meta-analysis combing EA and AA are listed in Table 3. Only two of these eleven SNPs were observed in AA. Therefore, the results of these SNPs in the meta-analysis were almost identical to those in the EA analysis. The only top EA SNPs observed in AA were the Chromosome 8 SNPs in the *WASCH5/SQLE* locus. While these two SNPs had minor allele frequencies of 6%–9% in EA, the frequencies were much higher in AA (42%–45%). The directions of associations with CVAE were consistent in AA patients compared to those in EA patients (Table 3).

**Table 3:** Top SNPs in the trans-ethnic meta-analysis of 247 ORIEN patients.

**CHR**	**SNP**	**BP**	**Gene**	**A1**	**A2**	**EA**				**AA**				**Meta-analysis**		
						**MAF**	**OR**	**95% CI**	***P***	**MAF**	**OR**	**95% CI**	***P***	**MAF**	**meta P**	**Direction**
2	rs7148	85,13,3216	TMSB10	A	G	0.068	9.33	3.90–22.35	5.42E-07	0	–	–	–	0.068	5.43E-07	+?
2	rs12,47,1929	85,13,3320	TMSB10	T	C	0.070	8.95	3.78–21.18	6.17E-07	0	–	–	–	0.070	6.18E-07	+?
5	rs28,45,0841	32,09,3210	PDZD2	T	C	0.077	5.25	2.38–11.58	3.91E-05	0	–	–	–	0.077	3.90E-05	+?
8	rs11,54,2889	12,60,44527	WASHC5/SQLE	T	C	0.059	5.01	2.23–11.24	9.50E-05	0.42	20.76	0.10–4300	0.27	0.067	5.67E-05	++
8	rs22,72,682	12,60,49443	WASHC5	C	T	0.094	3.83	1.95–7.49	9.26E-05	0.45	3.48	0.17–70.85	0.42	0.110	6.55E-05	--
5	rs22,91,113	32,07,4509	PDZD2	A	G	0.057	6.17	2.52–15.15	7.05E-05	0	–	–	–	0.057	7.06E-05	+?
4	rs17,38,7037	15,99,2783	PROM1	C	A	0.057	5.84	2.44–139.5	7.32E-05	0	–	–	–	0.943	7.31E-05	-?
2	rs75,34,8511	11,73,8951	GREB1	T	C	0.037	8.30	2.90–23.77	8.08E-05	0	–	–	–	0.037	8.08E-05	+?
5	rs37,33,720	32,08,7808	PDZD2	C	G	0.072	4.90	2.20–10.87	9.55E-05	0	–	–	–	0.072	9.55E-05	+?
5	rs10,06,6063	32,09,0294	PDZD2	A	G	0.072	4.90	2.20–10.87	9.55E-05	0	–	–	–	0.072	9.55E-05	+?
5	rs16,88,9442	32,09,3070	PDZD2	A	G	0.072	4.90	2.20–10.87	9.55 × 10^−5^	0	–	–	–	0.072	9.55E-05	+?

Chr., chromosome; SNP, single nucleotide polymorphism; EA, European American; AA, African Americans; A1, minor allele; A2, major allele; MAF, minor allele frequency; OR, odds ratio; CI, confidence interval.

### 3.3. Gene-based analysis

**Figure 3:**
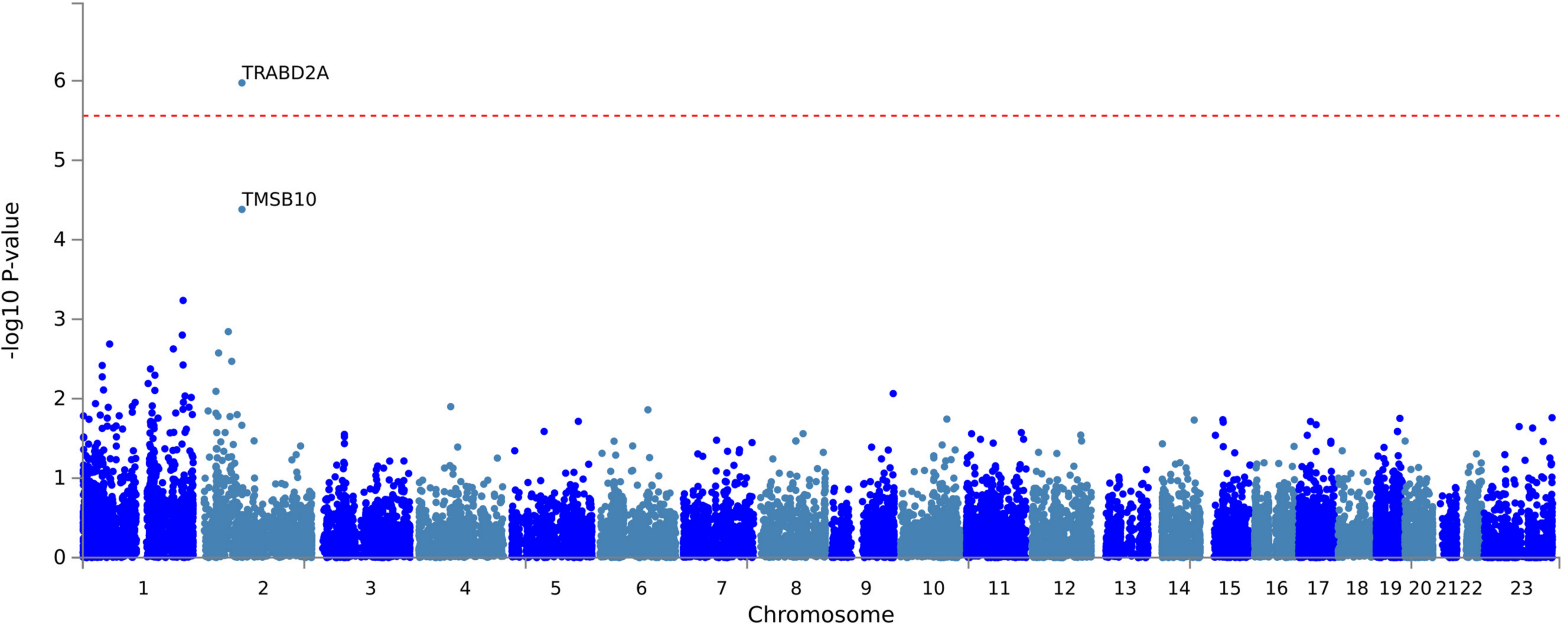
The gene-based association testing results from the FUMA analysis of CFZ-CVAE yielded *TRABD2A* as a significant gene (*p* = 1.06*10^−6^).

The gene-based analysis in EA patients using FUMA revealed that the *TRABD2A* gene that encodes TraB Domain Containing 2A is significantly associated with CFZ-CVAE (*p* = 1.06*10^−6^) (Figure 3).

### 3.4. Rare variant analysis performed using SKAT

SKAT analysis was performed on 40,969 gene sets and 620,661 SNPs, and a significant association was determined by comparing CFZ-CVAE after correcting for multiple testing. The genes—Chromosome 1 Open Reading Frame 116 (C1orf116), LOC102724084 (DYNLRB2 antisense RNA1), Diphosphoinositol pentakisphosphate kinase 2 (PPIP5K2 (NM_0013)), and Transmembrane Protein 183A (*TMEM183A*) were statistically significantly associated with CFZ-CVAE (*p*-value = 1.1*10^−5^, 4.1*10^−5^, 6.2*10^−5^, and 6.2*10^−5^, respectively) (Supplementary Table S6**)**.

### 3.5. IPA analysis of WES results

Using IPA, the pathway enrichment analysis showed that the lowest *p*-value and most significant genes overlapped with cardiotoxicity. The functional toxicity annotation of genes related to cardiotoxicity implicated cardiac arteriopathy with three variants from WES results: rs3750765 located in leucine-rich repeat-containing 20 (*LRRC20*) gene, rs72713436 in sterile alpha motif domain-containing 4A (*SAMD4A*) gene, and rs75454001 in CUB-Sushi multiple domains 1 (*CSMD1*) gene. All of these genes were associated with human coronary artery disease (*P* < 0.001) (28).

## 4. Discussion

In this first genetic association analysis of CFZ-related CVAE in MM patients, we conducted a WES of germline DNA samples from patients who received CFZ in the ORIEN network. In this retrospective cohort study of patients in a real-world clinical setting, we identified a missense SNP rs7148 in the *TMSB10/TRABD2A* gene locus on chromosome 2 to be associated with a higher risk for CVAE in MM patients treated with CFZ.

In order to reflect real-world practices, all MM patients treated with CFZ were included in this study regardless of their cardiovascular disease history. We found that 15.4% of the MM patients treated with CFZ developed CVAE, which is in line with the event rates of 8%–18% reported in prior meta-analyses (2, 3). Due to the retrospective nature of this study and a lack of clinical guidelines on proteome inhibitor monitoring at the time of this study, these patients were not actively monitored for cardiotoxicity by cardiologists. Not surprisingly, the event rate we observed is lower than the CFZ-CVAE event rate of 50% reported in a prospective study, the Prospective Observation of Cardiac Safety with Proteasome Inhibitor (PROTECT) study (10). The recently published 2022 Cardio-Oncology guideline by the European Society of Cardiology (29) recommends testing and surveillance of MM patients at baseline and every cycle during the first 6 cycles under proteasome inhibitor treatment based on the risk. The guideline recommends first stratifying MM patients into low, moderate, high, or very high-risk groups based on clinical factors prior to the treatment. Considering genetic variants may improve the risk stratification of these patients.

To our knowledge, this is the first genetic association study of CFZ-CVAE in humans. We found that the variant carriers of missense SNP rs7148 in the *TMSB10* gene were at higher risk for CFZ-CVAE. We also identified an intronic variant rs12471929 which is in perfect LD with rs7148 in EA. Both of these SNPs are also eQTLs for the nearby gene *TRABD2A* in that the variant alleles are associated with higher expression of TRABD2A in the left ventricle heart tissue (30).

Thymosins are a family of small peptides initially identified from the thymus and consist of three groups, alpha-, beta-, and gamma-thymosins, according to their isoelectric points (31). The beta-thymosins (TMSB4, TMSB10, TMSB15), found in the cytoplasm, interact with G-actin and produces a large pool of actin monomers (32). TMSB10 has been reported to function in cytoskeleton organization, cell morphology, proliferation, motility, and interaction with Ras and angiogenesis, cell growth, and apoptosis (33). The two isoforms, TMSB10 and TMSB4, are identified as significant actin monomer sequestering proteins that may regulate actin filament assembly (34). A recent study showed that the actin-binding protein TMSB10 was upregulated in dysfunctional endothelial dysfunction in acute myocardial infarction (AMI), and endothelial dysfunction is considered one of the primary factors in the progression of atherothrombosis in AMI (35). Another study on mice found that TMSB10 can inhibit vascular endothelial growth factor (VEGF) expression by inhibiting the Ras-ERK signaling pathway leading to the suppression of vascular formation, especially in tumor formation (36).

*TRABD2A* gene enables Wnt-protein binding activity and metalloendopeptidase activity (37). A recent genome-wide association study on cardiac troponin T (cTnT) in large cohorts identified a genetic variant rs548487604 near the *TRABD2A* gene to be associated with the elevation of cTnT level (38), which is related to the incidence of cardiovascular diseases, cardiovascular death, and heart failure in a general population (39, 40).

A previous study on mice proposed a pathway associated with CFZ-CVAE. This study found that treatment with CFZ induced the apoptosis pathway by activation of PP2A (protein phosphatase 2A), which inactivates AMPK*α* and the downstream signaling related to autophagy phosphoinositide 3′-kinase (PI3K)-Akt-endothelial nitric oxide synthase (eNOS) (PI3K/Akt/eNOS) axis. This axis is responsible for myocardial cell growth and survival and plays a vital role the cardiac dysfunction (41). Previous studies have identified a specific subtype of hematopoietic stem cells in peripheral blood called endothelial progenitor cells (EPCs) that express numerous combinations of antigens associated with hematopoietic stem and endothelial cells and play an essential role in neovascularization of ischemic tissue and reversal of endothelial dysfunction (42, 43), and *in vivo* studies showed that TMSB4 increases EPC migration and decreases EPC apoptosis under serum deprivation *via* the (PI3K/Akt/eNOS) signal transduction pathway (44, 45), and several studies showed that the telomerase length and telomerase activity of circulating EPCs and decreased in patients with coronary artery disease (46, 47). In a recent study that included 48 patients with relapsed/refractory MM and received CFZ, the brachial artery flow-mediated dilation (FMD) and 26s proteasome activity were detected to evaluate the endothelial function. This study concluded that patients who received CFZ and with low potential for proteasome activity recovery may suffer from both acute and long-term endothelial dysfunction (48).

Endothelial cell homeostasis depends on the ubiquitin-proteasome system, which induces oxidative stress in the cells and regulates the expression of endothelial nitric oxide synthase (49). The proteasome inhibitor CFZ causes the plasma of cancer cells, cardiomyocytes, and endothelial cells to accumulate with unfolded, dysfunctional proteins, which may lead to impaired vasodilation, excessive oxidative stress, inflammation, cell apoptosis, and autophagy (50, 51). Other studies have shown that the endothelial dysfunction caused by CFZ's inhibition of proteasome activity may result in CVAE or other endothelial dysfunction-related events like hypertension, heart failure, and coronary artery disease (51–53). In light of the literature, our finding of the genetic variants in the *TMSB10/TRABD2A* locus appears to support the role of endothelial dysfunction in CFZ-CVAE.

Among the other SNPs with a suggestive significance level, five are located on the PDZ domain-containing protein 2 (*PDZD2)* gene, which contains six PDZ domains and shares sequence similarity with pro-interleukin-16 (pro-IL-16). SNPs in the *PDZD2* gene have been associated with heart rate in heart failure patients with reduced ejection fraction (54). *PROM1* has a role in cell differentiation, proliferation, and apoptosis. PR1P is a small peptide derived from the extracellular domain of PROM1-derived peptide and improves cardiac function following ischemia. SQLE encodes squalene epoxidase, which catalyzes the first oxygenation step in sterol biosynthesis and is one of the rate-limiting enzymes in this pathway.

It is important to recognize some limitations of our study. Firstly, the patient population is predominantly European Americans. The sample size of patients of African descent was too small to have enough statistical power for any meaningful discovery. Further investigation is required to explore this phenotype and outcomes in MM patients of African ancestry. Secondly, using WES means we may have missed critical genetic variants outside the coding regions of the genome. Thirdly, using ICD codes to identify CVAE has its limitations. Even though our manual chart review of 10% of patients indicated 100% of CVAE were confirmed, it would have been ideal to review all the charts to confirm CVAE status. Fourthly, due to the small sample size, we had to combine all the CVAEs. Lastly, our study findings need to be replicated in an independent study before these genetic variants can be incorporated into the risk stratification of MM patients.

In summary, in this WES study of MM patients in a real-world clinical setting, we identified a missense variant in the *TMSB10/TRABD2A* locus to be associated with CFZ-CVAE among MM patients. Once validated, this association could provide the basis for a Precision Medicine approach to identify MM patients at high risk for CFZ-CVAE.

## Data Availability

The data presented in the study are deposited in the database of Genotypes and Phenotypes (dbGaP) repository, accession number phs003308.v1.p1.
