# Supplementary material for: Whole-Exome sequencing analysis identified TMSB10/TRABD2A locus to be associated with carfilzomib-related cardiotoxicity among patients with multiple myeloma
Source: Front Cardiovasc Med. 2023 Jun 20;10:1181806. doi: 10.3389/fcvm.2023.1181806 (PMC10319068; doi:10.3389/fcvm.2023.1181806)

Supplementary Material

TMSB10/TRABD2A locus associated with Carfilzomib-related cardiotoxicity in patients with Multiple Myeloma: Whole exome sequencing analysis.

Marwa Tantawy^1^, Guang Yang^2^, Raghunandan Reddy Algubelli^3^, Gabriel DeAvila^3^, Samuel M. Rubinstein^4^, Robert F. Cornell^5^, Michael G. Fradley^6^, Erin M. Siegel^7^, Oliver A. Hampton^8^, Ariosto S. Silva^9^, Daniel Lenihan^10^, Kenneth H. Shain^3^, Rachid C. Baz^3^, Yan Gong^1,11*^

*** Correspondence:** Yan Gong, Ph.D., MS., [gong@cop.ufl.edu](mailto:gong@cop.ufl.edu)

**Supplemental Table S1. List ICD9 and ICD10 codes.**

| ICD9 | ICD10 |
| --- | --- |
| 412 Old myocardial infarct | G72.9 Myopathy, unspecified |
| 422.90 Acute myocarditis NOS | I11.0 Hypertensive heart disease with heart failure |
| 422.91 Idiopathic myocarditis | I11.9 Hypertensive heart disease without heart failure |
| 422.93 Toxic myocarditis | I21.3 ST elevation (STEMI) myocardial infarction of unsp site |
| 425.4 Prim cardiomyopathy NEC | I24.9 Acute ischemic heart disease, unspecified |
| 425.9 Second cardiomyopathy NOS | I25.10 Athscl heart disease of native coronary artery w/o ang pctrs |
| 427.89 Cardiac dysrhythmias NEC | I25.2 Old myocardial infarction |
| 427.9 Cardiac dysrhythmia NOS | I25.5 Ischemic cardiomyopathy |
| 429.0 Myocarditis NOS | I42.0 Dilated cardiomyopathy |
| 429.3 Cardiomegaly | I42.2 Other hypertrophic cardiomyopathy |
| 785.0 Tachycardia NOS | I42.7 Cardiomyopathy due to drug and external agent |
| V15.1 Hx-major cardiovasc surg | I42.8 Other cardiomyopathies |
| V45.01 Status cardiac pacemaker | I42.9 Cardiomyopathy, unspecified |
|  | I43 Cardiomyopathy in diseases classified elsewhere |
|  | I49.8 Other specified cardiac arrhythmias |
|  | I49.9 Cardiac arrhythmia, unspecified |
|  | I50.20 Unspecified systolic (congestive) heart failure |
|  | I50.21 Acute systolic (congestive) heart failure |
|  | I50.30 Unspecified diastolic (congestive) heart failure |
|  | I50.32 Chronic diastolic (congestive) heart failure |
|  | I50.9 Heart failure, unspecified |
|  | I51.4 Myocarditis, unspecified |
|  | I51.89 Other ill-defined heart diseases |
|  | I51.9 Heart disease, unspecified |
|  | R00.9 Unspecified abnormalities of heart beat |
|  | R93.1 Abnormal findings on dx imaging of heart and cor circ |
|  | Z86.74 Personal history of sudden cardiac arrest |
|  | Z95.0 Presence of cardiac pacemaker |

**Supplemental Table S2. Demographics and clinical characteristics of European American MM patients in the ORIEN study.**

| Characteristics | Total  (n=228) | CVAEs  (n=35) | No CVAEs  (n=193) | P Value |
| --- | --- | --- | --- | --- |
| Age, year, mean ± SD | 59.5 ± 9.7 | 61.1 ± 9.3 | 59.2 ± 9.7 | 0.28 |
| Sex (Male) | 134 (58.8%) | 19 (54.3%) | 115 (59.6%) | 0.56 |
| Medical History |  |  |  |  |
| Hyperlipidemia | 71 (31.1%) | 14 (40.0%) | 57 (29.5%) | 0.22 |
| Hypertension | 117 (51.3%) | 23 (65.7%) | 94 (48.7%) | 0.064 |
| Diabetes | 21 (9.2%) | 5 (14.3%) | 16 (8.3%) | 0.26 |
| Ischemic Heart Disease | 5 (2.2%) | 1 (2.9%) | 4 (2.1%) | 0.77 |
| Myocardial Infraction | 11 (4.8%) | 3 (8.6%) | 8 (4.2%) | 0.26 |

**Supplemental Table S3. Other SNPs in LD with rs7148.**

| CHR | variant | LD (r2) | LD(D') | position (hg38) | A1 | A2 | dbSNP Function Annotation |
| --- | --- | --- | --- | --- | --- | --- | --- |
| 2 | rs7148 | 1 | 1 | 84906092 | A | G | Missense |
| 2 | rs12471929 | 1 | 1 | 84906196 | T | C | intronic |
| 2 | rs115246109 | 0.98 | 1 | 84907540 | C | G |  |
| 2 | rs139141384 | 0.98 | 1 | 84908733 | G | 11-mer |  |
| 2 | rs12473633 | 0.89 | 0.95 | 84909468 | C | T |  |

**Supplemental Table S4. Hardy Weinberg Equilibrium of the top SNPs in EA by CVAE status.**

| CHR | SNP | A1 | A2 | CVAE (Yes) (n=35) | | | | CVAE(No) (n=193) | | | |
| --- | --- | --- | --- | --- | --- | --- | --- | --- | --- | --- | --- |
|  |  |  |  | GENO | O(HET) | E(HET) | P (HWE) | GENO | O(HET) | E(HET) | P (HWE) |
| 2 | rs7148 | A | G | 1/14/20 | 0.400 | 0.353 | 0.651 | 0/15/178 | 0.078 | 0.075 | 1.000 |
| 2 | rs12471929 | T | C | 2/13/20 | 0.371 | 0.368 | 1.000 | 0/15/178 | 0.078 | 0.075 | 1.000 |
| 5 | rs28450841 | T | C | 2/11/22 | 0.314 | 0.337 | 0.631 | 0/20/173 | 0.104 | 0.098 | 1.000 |
| 5 | rs2291113 | A | G | 1/10/24 | 0.286 | 0.284 | 1.000 | 0/14/179 | 0.073 | 0.070 | 1.000 |
| 4 | rs17387037 | C | A | 1/10/24 | 0.286 | 0.284 | 1.000 | 0/14/179 | 0.073 | 0.070 | 1.000 |
| 2 | rs75348511 | T | C | 0/9/26 | 0.257 | 0.224 | 1.000 | 0/8/185 | 0.041 | 0.041 | 1.000 |
| 8 | rs2272682 | C | T | 4/10/21 | 0.286 | 0.382 | 0.177 | 2/21/170 | 0.109 | 0.121 | 0.177 |
| 8 | rs11542889 | T | C | 3/8/24 | 0.229 | 0.320 | 0.105 | 1/11/181 | 0.057 | 0.065 | 0.189 |
| 5 | rs3733720 | C | G | 2/10/23 | 0.286 | 0.320 | 0.593 | 0/19/174 | 0.098 | 0.094 | 1.000 |
| 5 | rs10066063 | A | G | 2/10/23 | 0.286 | 0.320 | 0.593 | 0/19/174 | 0.098 | 0.094 | 1.000 |
| 5 | rs16889442 | A | G | 2/10/23 | 0.286 | 0.320 | 0.593 | 0/19/174 | 0.098 | 0.094 | 1.000 |

CVAE: cardiovascular adverse events; A1: minor allele; A2: major allele; GENO: genotype counts for A1A1, A1A2, A2A2, respectively; O: observed; E: expected; HET: heterozygosity. HWE: Hardy Weinberg Equilibrium

**Supplemental Table S5. Summary WES analysis results of top variants associated with carfilzomib-CVAE in African American patients.**

| CHR | SNP | BP | Gene | A1 | A2 | MAF | OR | 95% CI | P | dbSNP functional annotation |
| --- | --- | --- | --- | --- | --- | --- | --- | --- | --- | --- |
| 4 | rs112075108 | 144435197 | SMARCA5-AS1 | T | C | 0.105 | 20.67 | 1.17-364.30 | 0.039 | synonymous |
| 17 | rs11079339 | 56270442 | EPX | G | A | 0.105 | 20.67 | 1.17-364.30 | 0.039 | missense |
| 5 | rs10050860 | 96122210 | ERAP1 | T | C | 0.132 | 18.28 | 1.09-305.80 | 0.043 | missense |
| 5 | rs2287987 | 96129535 | ERAP1 | C | T | 0.132 | 18.28 | 1.09-305.80 | 0.043 | missense |

**Supplemental Figure S1:** GTEx result on rs7148

Single-Tissue eQTLs P value= 1.9 x10^-7^


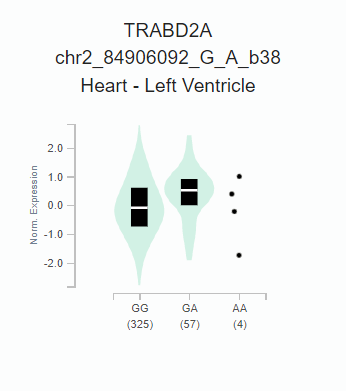

Supplement: Supplementary file 2 [file Table2.docx]
